# Angiopoietin-like protein 8 (ANGPTL8)/betatrophin overexpression does not increase beta cell proliferation in mice

**DOI:** 10.1007/s00125-015-3590-z

**Published:** 2015-04-28

**Authors:** Aaron R. Cox, Carol J. Lam, Claire W. Bonnyman, Julia Chavez, Jacqueline S. Rios, Jake A. Kushner

**Affiliations:** McNair Medical Institute, Baylor College of Medicine, Houston, TX USA; Section of Pediatric Diabetes and Endocrinology, Baylor College of Medicine, Houston, TX 77030 USA; Pediatric Diabetes and Endocrinology, Texas Children’s Hospital, Houston, TX USA

**Keywords:** Ageing, ANGPTL8, Betatrophin, Lipasin, Lipid homeostasis, Pancreatic beta cell, RIFL

## Abstract

**Aims/hypothesis:**

The identification of novel targets that stimulate endogenous regeneration of beta cells would represent a significant advance in the treatment of patients with diabetes. The betatrophin hypothesis suggests that increased expression of angiopoietin-like protein 8 (ANGPTL8) induces dramatic and specific beta cell proliferation and subsequent beta cell mass expansion with improved glucose tolerance. In light of recent controversy, we further investigated the effects of ANGPTL8 overexpression on beta cell proliferation.

**Methods:**

We performed hydrodynamic tail vein injections of green fluorescent protein (GFP) or *Angptl8* (also known as *Gm6484*) DNA in multiple cohorts of mice of different ages. We employed state-of-the-art methods to comprehensively quantify beta cell mass and proliferation, controlling for mouse age, genetic strain, source of DNA injected, *Angptl8* gene expression and proliferation markers.

**Results:**

In two young and two aged cohorts of B6.129 mice, no substantial change in beta cell replication, mass or glucose homeostasis was observed following ANGPTL8 overexpression. Even in mice with extremely elevated *Angptl8* expression (26-fold increase), beta cell replication was not significantly altered. Finally, we considered mice on the ICR background exactly as studied by Melton and colleagues, and still no beta cell mitogenic effect was detected following ANGPTL8 overexpression.

**Conclusion/interpretation:**

ANGPTL8 does not stimulate beta cell replication in young or old mice.

**Electronic supplementary material:**

The online version of this article (doi:10.1007/s00125-015-3590-z) contains peer-reviewed but unedited supplementary material, which is available to authorised users.

## Introduction

Diabetes is associated with reduced beta cell mass leading to an absolute (type 1) or relative (type 2) insulin deficiency and hyperglycaemia [1, 2]. Therapeutic islet transplantation suffers from loss of glucose control over time and scarcity of cadaveric islets. Stem cell generation of beta cells, while progressing, has failed to phenocopy human beta cells [3]. Consequently, the field of beta cell biology continues to focus on endogenous beta cell expansion. Normal beta cell growth occurs by self-duplication, without the contribution of specialised progenitor cells [4, 5]. Although putative beta cell mitogens have been advanced, none has progressed as a diabetes therapy [6].

Multiple studies suggest circulating factor(s) stimulate beta cell proliferation in response to increased metabolic demand [7–9]. Melton and colleagues administered insulin-receptor antagonist S961 to mice in search of circulating factors [10]. Significant beta cell expansion occurred following insulin receptor antagonism, and angiopoietin-like protein 8 (ANGPTL8) was highly upregulated in liver. ANGPTL8, also known as lipasin/RIFL, was previously recognised as a potent regulator of lipid metabolism [11–13]. Melton and colleagues overexpressed ANGPTL8 in the livers of ICR mice via hydrodynamic tail vein injection, inducing a beta cell replication rate ∼17-fold greater than controls, with a dramatic threefold expansion of beta cell area in 8 days and improved glucose tolerance [10]. They named the gene betatrophin, as it appeared that ANGPTL8 expanded beta cells via increased replication, furthering ANGPTL8 as a candidate therapy for diabetes. The discovery has been heralded as a major advance with the potential to improve human health [14–16]. Confusingly, ANGPTL8 levels are either increased or decreased in the serum of type 2 diabetes patients [17–19].

The actions of ANGPTL8 on beta cell generation have recently been called into question. Mice with knockout of *Angptl8* (also known as *Gm6484*) exhibited normal glucose homeostasis [20]. Kaestner and colleagues reported that S961 administration stimulated beta cell replication in mouse but not human islets, despite a fivefold increase in *Angptl8* mRNA in liver [21]. However, the failure of ANGPTL8 overexpression to induce beta cell replication in human islets in this study could have been a consequence of its use of a heterologous species model [22].

Gromada and colleagues reported that beta cell area was unaltered in *Angptl8*-knockout mice, even when challenged with a high-fat diet or treated with S961 [23]. They also suggest that ANGPTL8-overexpressing mice had no increase in beta cell mass, although measurements were highly variable, spanning a threefold range. Their data revealed a ∼30% increase in beta cell mass (the difference was not reported to be significant).

The Melton group published a reply providing data from 38 additional *Angptl8*-injected mice, asserting that some mice show a ‘jackpot’ effect on beta cell replication [24]. Although the magnitude of the response was smaller than previously observed, beta cell proliferation was still significantly above controls (*p* = 0.016). Notably, the variability of beta cell proliferation in ANGPTL8-overexpressing mice reported by the Melton group is entirely consistent with the variability of beta cell mass observed by Gromada and colleagues. Thus, Melton and colleagues propose a hypothesis in which the response to ANGPTL8 is highly variable, and some mice have a strong beta cell response. We aimed to definitively clarify the effects of ANGPTL8 on beta cell proliferation.

## Methods

### Mice

Male B6129SF1/J mice (JAX 101043) or retired male breeders (JAX 101045) were obtained from Jackson Labs (Bar Harbor, ME, USA). Male ICR mice were obtained from Taconic (Germantown, NY, USA). Intraperitoneal GTTs were performed on day 4 after a 16 h fast with 2 g d-glucose per kg. Insulin tolerance tests (ITTs) were performed on day 6 after a 4 h fast with 0.5–1.0 U insulin (Humulin R; Eli Lilly, Indianapolis, IN, USA) per kg. Mice were labelled continuously via the drinking water with 5-ethynyl-2′-deoxyuridine ([EdU]; 0.5 g/l; Life Technologies, Grand Island, NY, USA) from day 5 to 8. Mice were killed 8 days after injection for beta cell morphometric analysis. Random fed serum samples collected when the mice were killed were analysed for triacylglycerol, cholesterol and VLDL. Serum insulin was measured by Mouse Ultrasensitive Insulin ELISA kit (Alpco Diagnostics, Salem, NH, USA). B6.129 mice were fed 22% kJ from fat (catalogue number 2919; Harlan Laboratories, Houston, TX, USA). ICR mice were fed 16% kJ from fat (2920; Harlan Labs) [10].

### Hydrodynamic tail vein injection

B6.129 male mice of 2, 8 and 16 months of age or 7-week-old ICR male mice were used for hydrodynamic tail vein injections, as described previously [10, 25–28]. Green fluorescent protein (GFP) or *Angptl8* expression plasmid DNA (generous gifts from D. Melton, Harvard University [10]), 100 mg, and sleeping beauty transposase plasmid (pCMV-SB100X; Addgene, Cambridge, MA, USA), 4 mg, were injected as 8% of body weight volume (ml/g) over 5–7 s.

### Partial pancreatectomy

The splenic portion of pancreas was removed as previously [5, 29, 30], resulting in a ∼50% pancreatectomy (partial pancreatectomy [PPx]).

### Immunohistochemistry and morphometry

Paraffin sections for pancreatic head and tail were prepared as previously described [30, 31]. The entire pancreas was sectioned every 200 μm, generating 8–16 sections for both head and tail pancreas. Primary antisera included guinea pig anti-insulin (catalogue number A0564, Dako, Carpinteria, CA, USA) and mouse anti-human Ki67 (catalogue number 550609; BD Biosciences, San Jose, CA, USA), followed by secondary antisera conjugated to Cy3 or Cy5 (catalogue numbers 706-166-148 and 715-605-151; Jackson ImmunoResearch Laboratories, West Grove, PA, USA) and DAPI (Molecular Probes, Eugene, OR, USA). EdU was detected by Click-iT EDU Alexa Fluor 647 Imaging kit (Invitrogen, Carlsbad, CA, USA). Images were acquired using Zeiss AxioImager (Carl Zeiss MicroImaging, Thornwood, NY, USA) with Orca-ER digital camera (Hamamatsu, Middlesex, NJ, USA). Slides were imaged to quantify beta cell morphometry as previously described [30], using Volocity 6.1.1 software (PerkinElmer, Waltham, MA, USA).

### Proliferation analysis

At least 4,000 (1,700 for partial pancreatectomy) insulin^+^ cells were counted for Ki67. At least 2,900 (987 for partial pancreatectomy) insulin^+^ cells were counted for EdU. Ki67^+^ or EdU^+^ beta cell ratios were calculated as per cent total insulin^+^ cells.

### Real-time quantitative PCR

Total mRNA was extracted from liver using RNeasy Mini kit (Qiagen, Valencia, CA, USA) and cDNA prepared using High Capacity Reverse Transcription kit (Applied Biosystems, Foster City, CA, USA). Real-time quantitative (q) dual fluorescent-labelled fluorescence resonance energy transfer PCR (95°C 10 min, 40 cycles 95°C 15 s and 60°C 1 min) was performed with ABI ViiA7 real-time PCR system (Applied Biosystems) to amplify triplicate samples, comparing sample values with dilution curves. Relative gene product amounts are reported for each gene normalised to cyclophilin. Native (endogenous) *Angptl8* primer/probes distinguish from total *Angptl8* (exogenous plus native). Primers/probes (electronic supplementary material [ESM] Table 1) were purchased from Integrated DNA Technologies (Coralville, IA, USA). Results from a single experiment with technical replicates are averaged.

### Statistics

All data are mean ± SEM, unless otherwise stated. Results were compared with Student’s *t* test (unpaired) with a Bonferroni correction when appropriate, reported as *p* values.

### Study approval

The study was approved by the Baylor College of Medicine Institutional Animal Care and Use Committee.

## Results

### ANGPTL8 overexpression has powerful biological effects on lipids without any effect on glucose homeostasis in young B6.129 mice

**Fig. 1:**
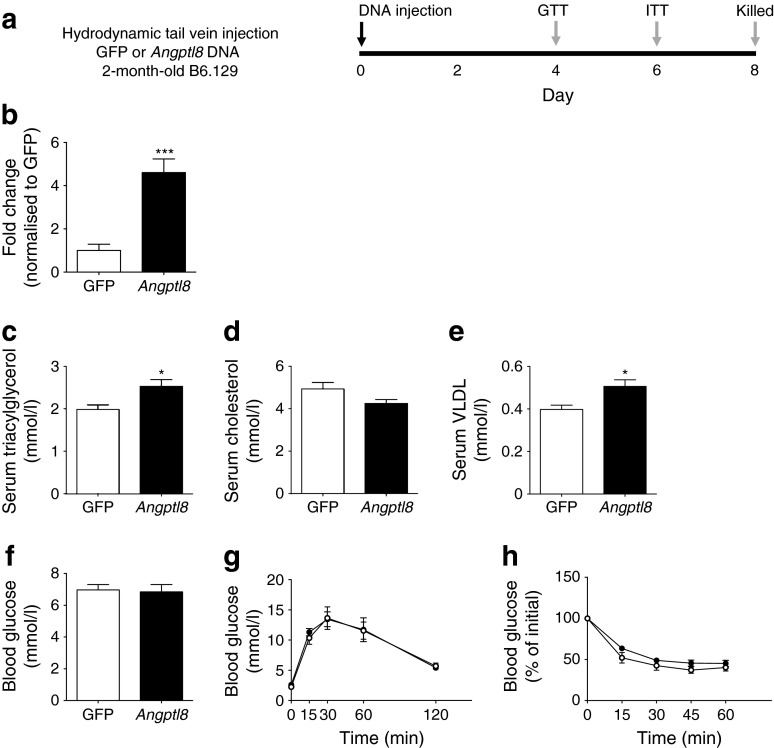
ANGPTL8 overexpression has powerful biological effects on lipids without any effect on glucose homeostasis in young B6.129 mice. (**a**) Schematic indicating time course of DNA injection, GTT, ITT and killing of 2-month-old B6.129 mice. (**b**) Total *Angptl8* gene expression analysis was performed by qPCR on liver samples, expressed as fold change from GFP-injected mice with cyclophilin used as control gene. (**c**–**f**) Measurements of random fed serum (**c**) triacylglycerol, (**d**) cholesterol, (**e**) VLDL and (**f**) blood glucose from GFP- or *Angptl8*-injected mice. (**g**) GTTs were performed on day 4, and (**h**) ITTs (represented as % of initial blood glucose) were performed on day 6, from GFP- and *Angptl8*-injected mice. Mean ± SEM, five animals per group. *p < 0.05 and ***p < 0.001 vs GFP. White, GFP-injected mice; black, *Angptl8*-injected mice

Melton and colleagues identified ANGPTL8 as a novel liver-derived protein that promotes beta cell growth [10]. We overexpressed ANGPTL8 via hydrodynamic tail vein injection in 2-month-old B6.129 mice (Fig. 1a). Total *Angptl8* gene expression increased 4.6-fold compared with GFP controls by qPCR (Fig. 1b, ESM Table 2), equivalent to the threefold induction of *Angptl8* expression induced by the insulin receptor antagonist S961 [10].

As ANGPTL8 was previously recognised as a potent regulator of lipid metabolism [11–13], we used serum lipid profiles as a biomarker of ANGPTL8 action. ANGPTL8 overexpression increased serum triacylglycerol and VLDL levels compared with GFP-injected controls (Fig. 1c, e), confirming biological activity of ANGPTL8. We then interrogated the impact of ANGPTL8 on glucose homeostasis. Overexpression of ANGPTL8 did not alter random fed blood glucose, glucose tolerance or insulin sensitivity (Fig. 1f–h; detailed physiological data are shown in ESM Table 3). Despite a powerful effect on lipids, ANGPTL8 had no effect on glucose homeostasis.

### ANGPTL8 overexpression does not alter beta cell area in young B6.129 mice

**Fig. 2:**
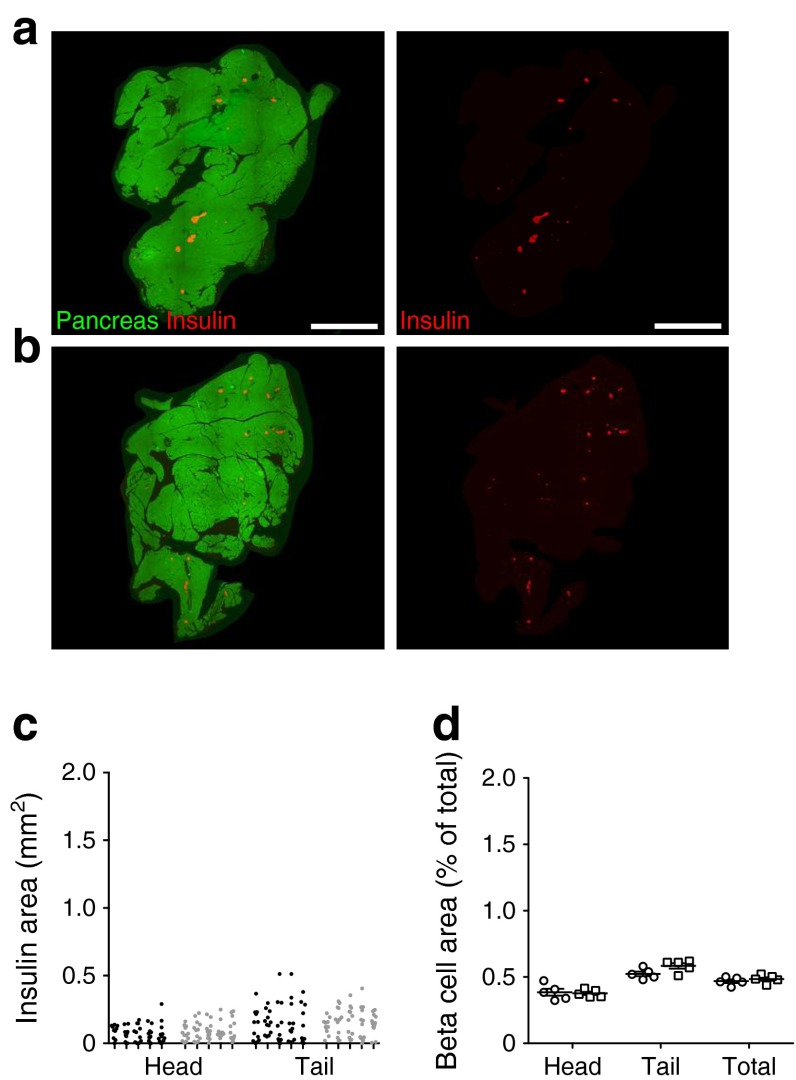
ANGPTL8 overexpression does not alter beta cell area. (**a**, **b**) Representative low-power images of total pancreas (green) and total insulin area (red) for (**a**) GFP and (**b**) *Angptl8* DNA-injected 2-month-old B6.129 mice. Scale bars: 2 mm. (**c**) For each mouse we analysed 10–12 pancreatic sections per head or tail region, and plotted the cross-sectional insulin area for each pancreatic section as a single dot within a vertical column. Black dots, GFP; grey dots, *Angptl8*. (**d**) Beta cell area (% of total pancreas) for the head, tail and total pancreas. Circles, GFP; squares, *Angptl8*. Mean ± SEM, five animals per group

We used high-throughput quantitative imaging methods to examine beta cell content throughout the entire pancreas, sampling every 200 μm to obtain total pancreas and insulin area (Fig. 2a, b). ANGPTL8 overexpression did not increase the cross-sectional insulin area or beta cell area (% of total) compared with the GFP-injected group in young B6.129 mice (Fig. 2c, d, ESM Table 4). These results contrast with observations of a dramatic threefold increase in beta cell area over the same time period reported by Melton and colleagues [10].

### ANGPTL8 overexpression does not alter beta cell proliferation in young B6.129 mice

**Fig. 3:**
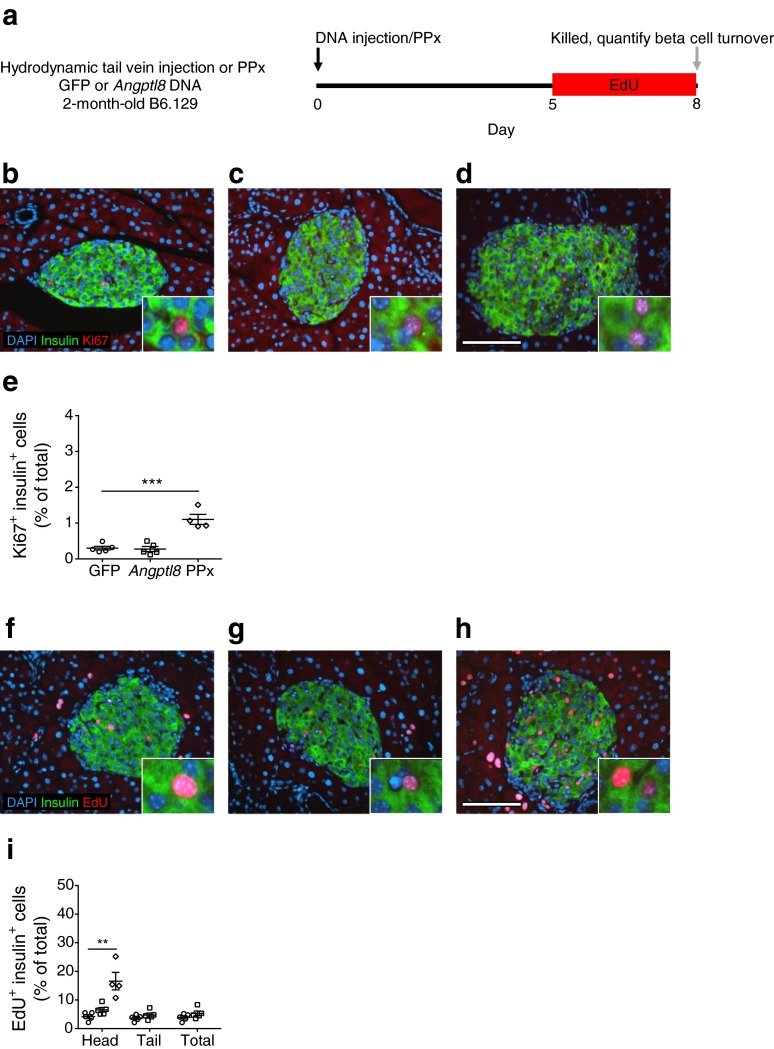
ANGPTL8 overexpression does not alter beta cell proliferation in young B6.129 mice. (**a**) Schematic indicating time course for DNA injection or PPx, with EdU labelling from day 5 to 8. Immunostaining for (**b–d**) Ki67 (red), insulin (green) and DAPI (blue) for (**b**) GFP, (**c**) *Angptl8* and (**d**) PPx groups (2-month-old B6.129 mice). (**e**) Quantification of beta cell proliferation measured by Ki67^+^ insulin^+^ cells as a percentage of total insulin^+^ cells for total pancreas. Immunostaining for (**f–h**) EdU (red), insulin (green) and DAPI (blue) for (**f**) GFP, (**g**) *Angptl8* and (**h**) PPx groups (2-month-old B6.129 mice). (**i**) Quantification of beta cell proliferation measured by EdU^+^ insulin^+^ cells as a percentage of total insulin^+^ cells in the head, tail and total pancreas. Scale bars: 100 μm. Mean ± SEM, four to five animals per group. **p < 0.01 and ***p < 0.001 vs GFP

Despite having no effect on beta cell area, ANGPTL8 may still modestly increase proliferation as recently reported [24]. Thus, we quantified beta cell proliferation via Ki67 to determine if ANGPTL8 overexpression impacts beta cell replication (Fig. 3).

Beta cells expressing Ki67 were rare in both GFP- and *Angptl8*-injected mice (Fig. 3b, c). In contrast, Ki67^+^ insulin^+^ cells were dramatically increased following partial pancreatectomy ([PPx]; Fig. 3d, ESM Table 5), a well-established model of beta cell proliferation [29, 30]. Quantification of Ki67^+^ beta cell proliferation showed no effect of ANGPTL8 overexpression on replication, while PPx induced a threefold increase over GFP controls (Fig. 3e).

Ki67 status gives a snapshot in time of beta cell replicative events. Thus, we also performed thymidine analogue (EdU) labelling over the final 3 days (Fig. 3a). Equally low levels of EdU incorporation were observed in beta cells of GFP and *Angptl8* groups (Fig. 3f, g). ANGPTL8 overexpression had no effect on the percentage of EdU^+^ beta cells, although proliferation was significant in the head pancreases of partially pancreatectomised mice (Fig. 3f–i, ESM Table 5). These results indicate that ANGPTL8 does not increase beta cell replication in young B6.129 mice, especially in relation to PPx, a potent inducer of beta cell proliferation.

### ANGPTL8 expression in young mice

We considered that our findings could be confounded by using the wrong strain/age of mice or achieving an insufficient level of ANGPTL8 overexpression. Consequently we expanded our analysis to include a second cohort of 2-month-old B6.129 mice, two additional aged B6.129 cohorts (8- and 16-month-old), and young ICR mice (the exact strain and source of mice employed by Melton and colleagues [10]). Our initial qPCR primer/probe set measured total *Angptl8* (exogenous and endogenous), thus we developed tools to quantify native (endogenous) *Angptl8* gene expression.

**Fig. 4:**
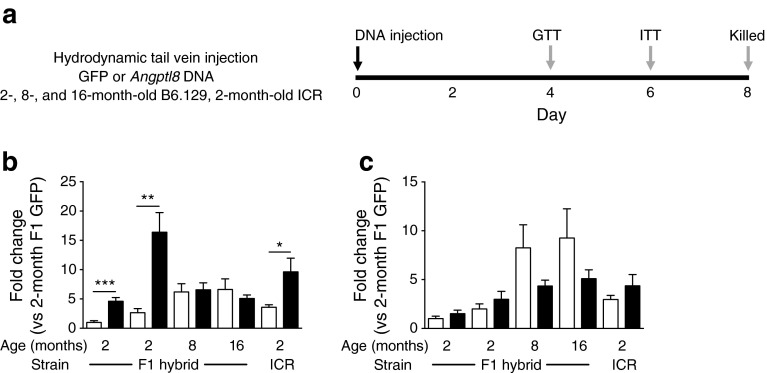
Native *Angptl8* increases with age and is reduced in aged mice following overexpression of exogenous *Angptl8*. (**a**) Schematic indicating time course for two cohorts of 2-, 8- and 16-month-old B6.129 mice and 2-month-old ICR mice. (**b**) Total and (**c**) native *Angptl8* gene expression analysis was performed by qPCR on liver samples, expressed as fold change from original 2-month-old GFP-injected B6.129 mice with cyclophilin used as control gene. The original 2-month B6.129 cohort is listed first in each graph (furthest left) followed by a second 2-month cohort. Mean ± SEM, five to seven animals per group. *p < 0.05, **p < 0.01 and ***p < 0.001

Total *Angptl8* expression was increased sixfold in the second cohort of 2-month B6.129 mice compared with GFP (Fig. 4b). This increase in expression was threefold higher than the original cohort of *Angptl8*-injected mice and 16-fold in absolute *Angptl8* mRNA levels compared with controls for our first cohort. Native *Angptl8* mRNA expression was unchanged by ANGPTL8 overexpression in both cohorts of 2-month-old B6.129 mice, indicating that the exogenous *Angptl8* entirely contributed to the increase in total *Angptl8* (Fig. 4b, c, ESM Table 2).

We also examined the impact of ANGPTL8 overexpression in young ICR mice. Total *Angptl8* was increased by 3.2-fold compared with GFP, with no change in native *Angptl8* (Fig. 4b, c). Thus, we were able to overexpress *Angptl8* in the exact strain of mice employed by Melton and colleagues [10].

### ANGPTL8 overexpression reduces native *Angptl8* expression in aged mice

We injected GFP or *Angptl8* into 8- and 16-month-old B6.129 mice to examine the age-related impact of exogenous *Angptl8* overexpression. Control aged mice exhibited ∼tenfold increase in native *Angptl8* expression compared with mice at 2 months (*p* < 0.05; Fig. 4c, ESM Table 2). Surprisingly, total *Angptl8* expression was unchanged in aged mice injected with *Angptl8*, while native *Angptl8* expression was reduced (Fig. 4b, c). This result suggests that counter-regulatory feedback loops preserve *Angptl8* expression at a constant level in older mice. If such counter-regulatory loops protect *Angptl8* expression levels, this could challenge the potential therapeutic utility of interventions to augment *Angptl8* expression in type 2 diabetes.

### ANGPTL8 overexpression does not alter beta cell area in B6.129 mice of various ages or in ICR mice

We performed extensive morphometric analysis of the entire pancreas using high-throughput image processing to quantify beta cells (ESM Figs 1 and 2, ESM Table 4). Insulin area, quantified as cross-sectional or cumulative total, was largely unchanged by ANGPTL8 overexpression in B6.129 mice at any age or in ICR mice (ESM Fig. 2a–h). A minimal increase in insulin area was observed in the tail of the additional 2-month-old B6.129 group (ESM Fig. 2e). Given the tiny magnitude of this difference, it likely represents the precision with which our techniques can detect small changes in biological variation as opposed to the physiological intervention of ANGPTL8 overexpression. Cumulative total pancreas area was unaltered (ESM Fig. 2i–l).

**Fig. 5:**
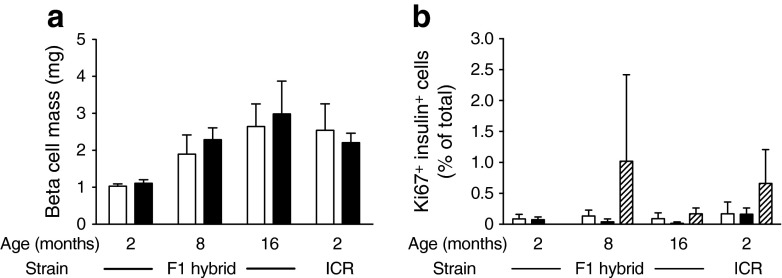
ANGPTL8 overexpression does not alter beta cell mass or proliferation in B6.129 mice of various ages or ICR mice. (**a**) Total beta cell mass and (**b**) total beta cell proliferation measured as Ki67^+^ insulin^+^ cells (% of total insulin^+^ cells) in GFP, *Angptl8* and PPx groups. Cohorts include a second group of 2-month-old B6.129 mice, 8- and 16-month-old B6.129 mice and 2-month-old ICR mice. Mean ± SD, five to eight animals per group. White bars, GFP; black bars, *Angptl8*; hatched bars, PPx

Total pancreatic beta cell area and mass were equivalent between GFP and ANGPTL8 for all four cohorts (Fig. 5a, ESM Fig. 2m–t). Thus, we were unable to confirm prior reports [10] of massive beta cell expansion by ANGPTL8 in our studies with multiple cohorts of mice at various ages, including strain-matched young ICR mice.

### ANGPTL8 overexpression does not alter beta cell proliferation in B6.129 mice of various ages or in ICR mice

Beta cell proliferation in the additional cohort of 2-month-old B6.129 mice (which expressed extremely high levels of *Angptl8*) was unaltered by Ki67 or EdU (Fig. 5b, ESM Figs 3 and 4, ESM Table 5). Beta cell proliferation was equivalent between GFP and ANGPTL8 groups in older mice. As expected, beta cell proliferation was potently increased by PPx in 2- and 8-month-old mice. ANGPTL8 overexpression had no effect on beta cell proliferation in young ICR mice, unlike PPx. Thus, we conclude that ANGPTL8 does not stimulate beta cell replication.

## Discussion

Although Melton and colleagues recently asserted that ANGPTL8 overexpression variably increases beta cell proliferation, we show here that ANGPTL8 overexpression in mice does not alter beta cell proliferation. We attempted to replicate the original and additional ANGPTL8 overexpression studies [10, 23, 24], including multiple cohorts of mice quantified with advanced imaging modalities. We carried out rigorous analysis of beta cell replication with both Ki67 and EdU, demonstrating that ANGPTL8 does not stimulate beta cell proliferation.

Mouse genetic strain and age could influence putative beta cell trophic actions of ANGPTL8, therefore we included an array of strains and ages. Moreover, we used the Melton *Angptl8* plasmid [10]. Our initial studies in 2-month-old B6.129 mice suggested that ANGPTL8 overexpression does not expand beta cells. We then overexpressed ANGPTL8 in a second cohort of 2-month-old B6.129 mice. For reasons that are unclear, the second cohort had absolute *Angptl8* levels that vastly exceeded other cohorts (up to 26-fold that of controls in two mice). This additional cohort therefore allowed us to rigorously test the betatrophin hypothesis, assuming higher *Angptl8* expression might lead to greater stimulation of beta cell area or proliferation. However, even extreme *Angptl8* expression had no effect on beta cell expansion.

Control mice at 8 and 16 months of age had higher levels of *Angptl8* expression than young control mice. However, aged mice, despite their higher levels of *Angptl8* expression, had far less beta cell proliferation than younger mice. Thus, even our aged controls challenge the betatrophin hypothesis.

Last, we considered the possibility that genetic strain differences could explain the lack of response in B6.129 mice. We administered *Angptl8* to young ICR male mice, as performed by Melton and colleagues [10], but again found no change in beta cell proliferation. Gromada and colleagues did not match the Melton strain, age and DNA, leaving their results open to criticism that unforeseen variables could have influenced the regenerative response [23]. The reason for the discrepancy between our results and those of Melton and colleagues is not clear. However, here we have controlled for experimental conditions, matching those used by the Melton group, and conclude that ANGPTL8 overexpression has no effect on beta cell proliferation or area.

Insufficient or inappropriate sampling of beta cells could introduce unwanted variability and bias, rendering the subsequent data uninterpretable. To avoid erroneous sampling from regions of the pancreas rich in beta cells, we divided the pancreas into head and tail, sectioning every 200 μm from top to bottom. This method resulted in 8–16 sections per pancreas portion, allowing for a comprehensive analysis of pancreatic beta cell area (complete raw data are in ESM Table 4).

We were concerned about the possibility of proliferating intra-islet non-beta cells contaminating our process to quantify beta cell proliferation (as previously examined [30]). As a result, we exclusively imaged islets with a ×40 high numerical aperture objective (0.70) to ensure beta cells (and not other cells) were correctly identified within islets. As proliferative events marked by Ki67 may be rare and highly variable, we counted at least 4,000 beta cells per pancreas in GFP- and *Angptl8*-injected mice (complete raw data in ESM Table 5). Similarly, we counted over 2,900 beta cells to quantify EdU-labelled beta cells. Thus, we are confident that we have correctly assessed any potential changes in beta cell area/proliferation, and demonstrate minimal variability in beta cell area/proliferation across all cohorts, with no significant change in ANGPTL8-overexpressing mice.

Our studies included PPx as a positive control for beta cell proliferation in order to put the putative trophic effect of ANGPTL8 in the context of other known beta cell mitogenic stimuli. PPx is well documented to induce beta cell proliferation [29, 30]. As expected, PPx induced beta cell replication by three- to fivefold in young B6.129 and ICR mice. However, ANGPTL8 overexpression had no effect on beta cell replication. A regenerative response to PPx was maintained at 8 months of age but declined by 16 months, corroborating previous studies demonstrating an age-related decline in adaptive beta cell proliferation [29]. Even though beta cells remained responsive to regenerative stimuli at 8 months of age, no such response was observed with ANGPTL8 overexpression in mice at 8 or 16 months.

The large number of biological replicates allows us to test the ‘jackpot’ hypothesis recently advanced by Melton and colleagues [24], whereby some mice respond strongly to ANGPTL8 but many others do not. In this scenario, occasional mice would have extreme beta cell proliferation compared with their peers because of intrinsic differences caused by some unidentified variable. To address the possibility of a variable jackpot response, we carried out our studies in a large number of mice (28 controls and 31 experimental animals). We quantified beta cell proliferation with Ki67 (as performed by Melton and colleagues with great variability) and long-term EdU labelling to aid precise measurement of beta cell proliferation. However, no proliferative response to ANGPTL8 expression was observed, with little intrinsic variation in beta cell proliferation within mice. The largest variability occurred in the original 2-month-old B6.129 cohort; beta cell proliferation in one mouse was 0.5% with Ki67 while another mouse was 0.12% (ESM Table 5). Young ICR *Angptl8*-injected mice had EdU^+^ replication rates that peaked at 7.67% and 7.29% (identified as 325.1 W and 325.4 W, respectively), which was comparable with the highest beta cell proliferation in the control GFP group (7.17%, identified as 323.3 W; ESM Table 5).

The lack of intrinsic variability in beta cell response to ANGPTL8 is in sharp contrast to the highly variable beta cell proliferation results of Melton and colleagues, and the variable beta cell mass observed by Gromada and colleagues [10, 23, 24]. The absolute values and equally small variability across all cohorts are inconsistent with the hypothesis that a subset of mice potently respond to ANGPTL8 overexpression. Instead, it seems likely that any variation previously attributed to contribute to a jackpot hypothesis might be the result of under-sampling or technical differences in between cohorts (such as variable age of mice tested). Taken together, these results strongly oppose the premise that some mice respond strongly to ANGPTL8 overexpression.

A final potential confounding factor to consider is the expression level of *Angptl8* necessary to induce beta cell proliferation. Previous reports did not quantify *Angptl8* gene expression following hydrodynamic tail vein injection [10, 23, 24]. Liver *Angptl8* was increased by ∼4.5-fold in NOD severe combined immunodeficiency (SCID) mice and threefold in ICR mice following 7 days of S961 treatment and was associated with an extraordinary amount of beta cell proliferation (>20- and 12-fold) [10, 21]. These expression values are equivalent to those found in our studies, with total *Angptl8* increasing by 3.2-fold and 4.6-fold in ICR and B6.129 mice, respectively. Importantly, our second cohort of *Angptl8*-injected mice had extremely high levels of *Angptl8* expression (sixfold vs GFP and 16-fold compared with controls of our first cohort), but no increase in beta cell proliferation.

In summary, we show that ANGPTL8 overexpression does not increase beta cell proliferation. Melton and colleagues initially reported that exogenous *Angptl8* induced massive beta cell expansion by threefold within 8 days [10]. Gromada and colleagues recently reported that ANGPTL8 does not control beta cell expansion [23]. Notably, Gromada and colleagues did not quantify beta cell proliferation. Confusingly, Melton and colleagues responded that ANGPTL8 overexpression increased beta cell replication [24]. However, we provide studies in multiple cohorts of mice of various ages and genetic strains, employing the most rigorous methods in current use to quantify beta cells, finding that ANGPTL8 overexpression does not alter beta cell proliferation or expansion.

## Electronic supplementary material

ESM Fig. 1 (PDF 402 kb)

ESM Fig. 2 (PDF 346 kb)

ESM Fig. 3 (PDF 466 kb)

ESM Fig. 4 (PDF 133 kb)

ESM Table 1 (PDF 54 kb)

ESM Table 2 (XLSX 117 kb)

ESM Table 3 (XLSX 97 kb)

ESM Table 4 (XLSX 102 kb)

ESM Table 5 (XLSX 157 kb)

## Abbreviations

ANGPTL8: Angiopoietin-like protein 8
EdU: 5-Ethynyl-2′-deoxyuridine
GFP: Green fluorescent protein
ITT: Insulin tolerance test
PPx: Partial pancreatectomy
