# Supplementary material for: Angiopoietin-like protein 8 (ANGPTL8)/betatrophin overexpression does not increase beta cell proliferation in mice
Source: Diabetologia. 2015 Apr 28;58(7):1523–31. doi: 10.1007/s00125-015-3590-z (PMC4473078; doi:10.1007/s00125-015-3590-z)
Supplement: Supplementary file 1 — (PDF 402 kb) [file 125_2015_3590_MOESM1_ESM.pdf]

ESM Fig. 1

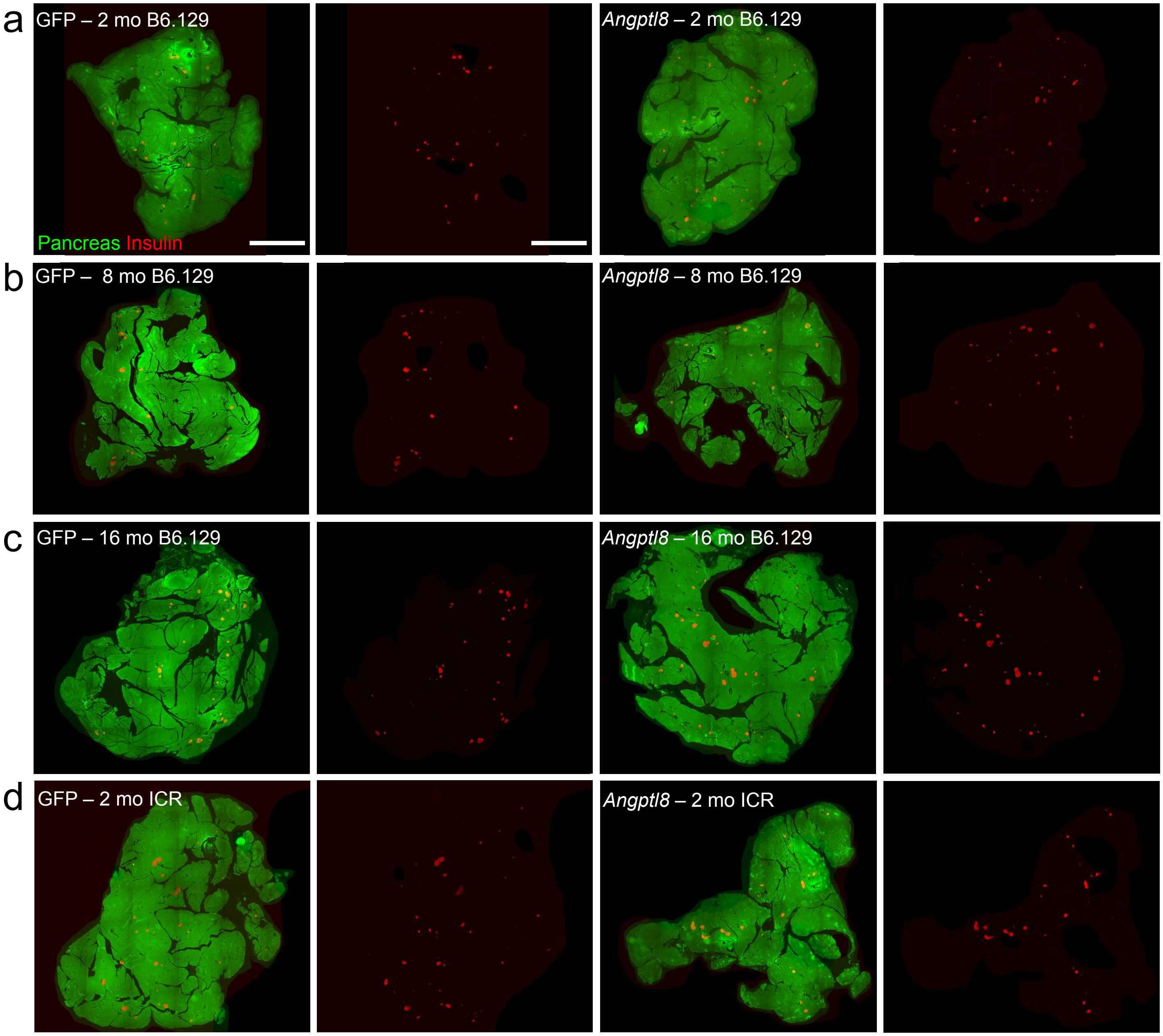

ESM Fig. 1. Representative images of GFP- and *Angptl8*-injected mice at various ages. Representative low-power images of total pancreas (green) and total β-cell area (red) for GFP and ANGPTL8 DNA-injected mice from **(a)** the second cohort of 2-month-old B6.129 mice, **(b)** 8- and **(c)** 16-month-old F1 hybrid mice, and **(d)** 2-month-old ICR mice. Scale bars: 2 mm.
