# Supplementary material for: Angiopoietin-like protein 8 (ANGPTL8)/betatrophin overexpression does not increase beta cell proliferation in mice
Source: Diabetologia. 2015 Apr 28;58(7):1523–31. doi: 10.1007/s00125-015-3590-z (PMC4473078; doi:10.1007/s00125-015-3590-z)
Supplement: Supplementary file 2 — (PDF 346 kb) [file 125_2015_3590_MOESM2_ESM.pdf]

ESM Fig. 2

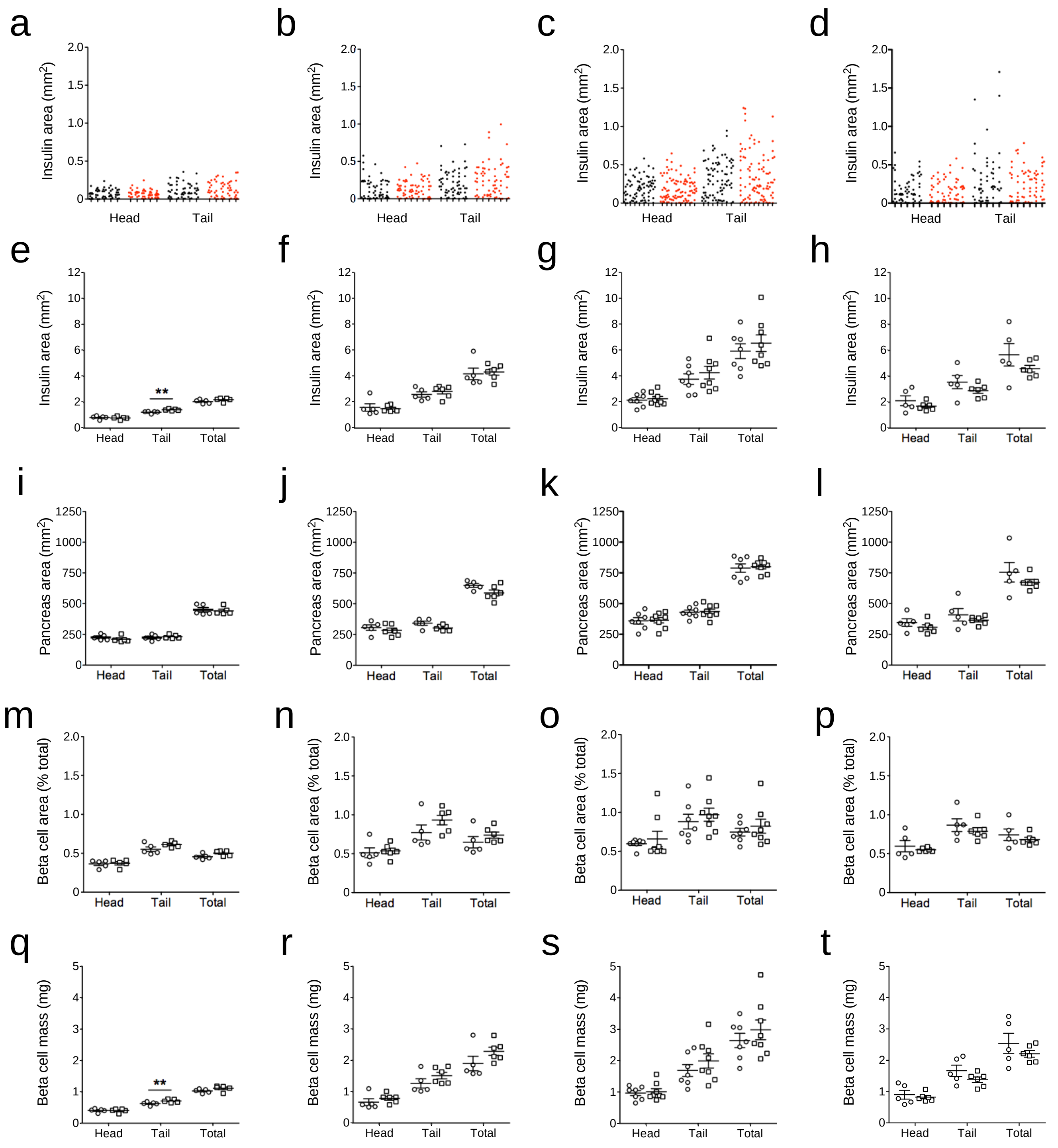

ESM Fig. 2. Extensive analysis of β-cell area in B6.129 mice of various ages or ICR mice. Cohorts include a second independent group of 2-month B6.128 mice, 8- and 16-month-old B6.129 mice, and 2-month-old ICR mice. Pancreatic sections were obtained every 200 µm through the entire pancreas, head and tail (8-16 sections analyzed for each head and tail portion). **(a-d)** Cross-sectional insulin area (mm^2) for each individual mouse plotted in a vertical column with each dot representing analysis of a single pancreatic cross section. Black dots, GFP; red dots, *Angptl8*. **(e-l)** Cumulative total **(e-h)** insulin and **(i-l)** pancreas area (mm^2) for all cross sections analyzed. **(m-p)** β-cell area (% of total pancreas area) and **(q-t)** β-cell mass (mg) for the head, tail, and total pancreas. Circles, GFP; squares, *Angptl8*. Data represent mean ± SEM, with five to eight animals per group. **p<0.01
