# Supplementary material for: Angiopoietin-like protein 8 (ANGPTL8)/betatrophin overexpression does not increase beta cell proliferation in mice
Source: Diabetologia. 2015 Apr 28;58(7):1523–31. doi: 10.1007/s00125-015-3590-z (PMC4473078; doi:10.1007/s00125-015-3590-z)
Supplement: Supplementary file 3 — (PDF 466 kb) [file 125_2015_3590_MOESM3_ESM.pdf]

ESM Fig. 3

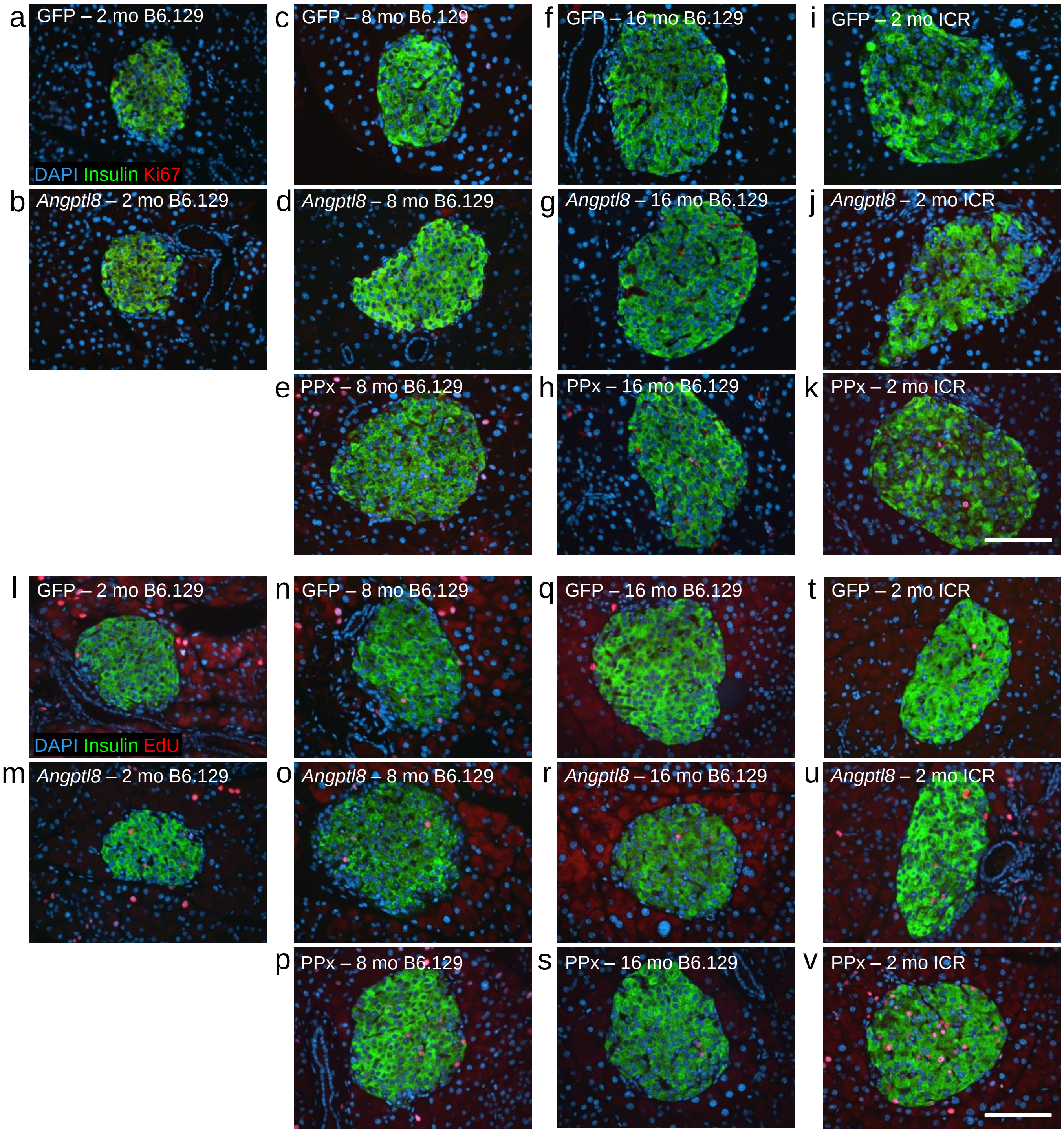

ESM Fig. 3. Representative images of β-cell proliferation from GFP, *Angptl8*, and PPx groups at various ages. Representative images of insulin (green), DAPI (blue) and **(a-k)** Ki67 (red) or **(l-v)** EdU (red) for GFP- (left column) and *Angptl8*-injected (middle) mice, or partial pancreatectomized mice (right). Images are from **(a-b, l-m)** the second cohort of 2-month-old B6.129 mice, **(c-e, n-p)** 8- and **(f-h, q-s)** 16-month-old B6.129 mice, and **(i-k, t-v)** 2-month-old ICR mice. Scale bars: 100 µm.
