# Supplementary material for: Angiopoietin-like protein 8 (ANGPTL8)/betatrophin overexpression does not increase beta cell proliferation in mice
Source: Diabetologia. 2015 Apr 28;58(7):1523–31. doi: 10.1007/s00125-015-3590-z (PMC4473078; doi:10.1007/s00125-015-3590-z)
Supplement: Supplementary file 4 — (PDF 133 kb) [file 125_2015_3590_MOESM4_ESM.pdf]

ESM Fig. 4

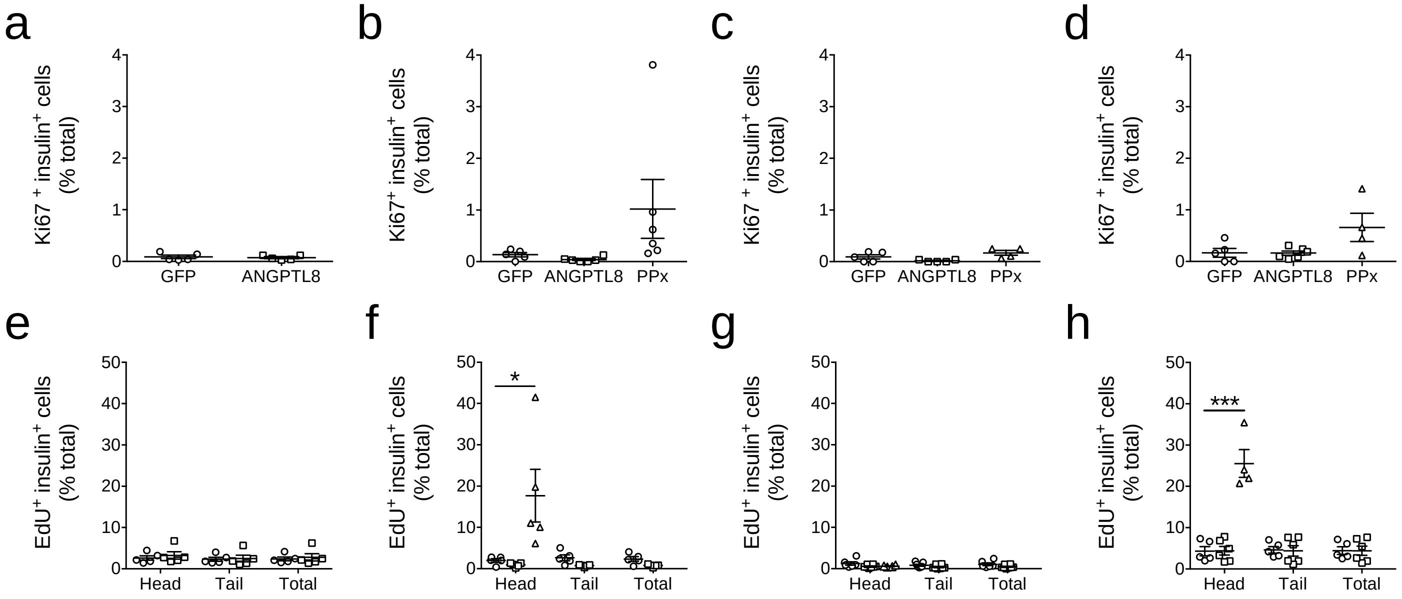

ESM Fig. 4. ANGPTL8 overexpression does not alter β-cell proliferation in B6.129 mice of various ages or ICR mice. β-cell proliferation was measured in GFP (circles), *Angptl8* (squares), and PPx (triangles) groups for the **(a,e)** second independent cohort of 2-month-old B6.129, **(b,f)** 8- and **(c,g)** 16-month-old B6.129, and **(d,h)** 2-month-old ICR mice. **(a-d)** Ki67$^+$ insulin$^+$ cells as a percentage of total β-cells for the total pancreas and **(e-h)** EdU$^+$ insulin$^+$ cells as a percentage of total β-cells in the head, tail, and total pancreas. Data represent mean ± SEM, four to eight animals per group. *p<0.05, ***p<0.001 versus GFP.
