# Supplementary material for: Angiopoietin-like protein 8 (ANGPTL8)/betatrophin overexpression does not increase beta cell proliferation in mice
Source: Diabetologia. 2015 Apr 28;58(7):1523–31. doi: 10.1007/s00125-015-3590-z (PMC4473078; doi:10.1007/s00125-015-3590-z)
Supplement: Supplementary file 5 — (PDF 54 kb) [file 125_2015_3590_MOESM5_ESM.pdf]

ESM Table 1. Primer and probe set sequences.

Gene	Forward	Reverse	Probe
**Total *Angptl8***	ATTCCTGGGGACAGAAGTCA	GCTTTACACCTTCGAGCTGA	TGCCACACAGGAGCTTCGCA
**Native *Angptl8***	AGCAGAGCCACCTCTTATGG	TGAGACTACCTGGATGCCAC	CACACAGCAGCCCTCCCAGC
**Cyclophilin**	CAGACGCCACTGTCGCTTT	TTGCAGACAAAGTTCCAAAGACA	CCCTTGGGCCGCGTCTCCTT
